# Predicting Leadership Competency Development and Promotion Among High-Potential Executives: **The** Role of Leader Identity

**DOI:** 10.3389/fpsyg.2020.01816

**Published:** 2020-08-05

**Authors:** Darja Kragt, David V. Day

**Affiliations:** ^1^School of Psychological Science, The University of Western Australia, Perth, WA, Australia; ^2^Kravis Leadership Institute, Claremont McKenna College, Claremont, CA, United States

**Keywords:** leadership development, leader identity, high-potential executives, leadership competencies, promotion

## Abstract

We propose that distinct leadership competencies differ in their development over time. Extending the integrative model of leader development (Day et al., 2009), we further propose that leader identity will form complex relationships with leadership competencies over time. To test these propositions, we use longitudinal data (i.e., 5 months, four measurement points) of the 80 in total high-potential executives in a corporate leadership development program. We find a significant difference in the initial levels and the changes of eight distinct leadership competencies. We also find that leader identity relates to the development of certain – but not all – leadership competencies. Finally, we demonstrate the importance of developing leadership competencies by linking them to career advancement (i.e., job promotion). These findings are discussed in light of their theoretical and practical implications.

## Introduction

Leadership competency models are widely used by organizations and practitioners in targeting their leader development efforts. Despite a debate about the value of leadership competencies in leader development and talent management efforts (Hollenbeck et al., 2006), the complexity of a senior leadership role involves a number of diverse competencies. Mapping change at the individual-leader level benefits from the use of a competency approach to better understand what changes as a function of a developmental initiative and what form the changes take. Thus, we model the development of eight different leadership competencies in a 5-month leadership development program. We also study a time-varying predictor (i.e., leader identity) and career outcome (i.e., job promotion) associated with leadership competency development. We propose that the development of these competencies is driven by the acquisition of new leadership-related knowledge and through practice, both facilitated through participation in a leadership development program as well as activities undertaken outside the program (e.g., Mumford et al., 2000a; Day et al., 2009). It is also the case that at higher organizational levels, leaders require a more complex set of competencies to fulfill the growing demands of their roles (Mumford et al., 2007; Dragoni et al., 2011). Nonetheless, there is little evidence about how leadership competencies develop across time as part of a corporate leadership development initiative. In this manner, we extend previous literature by examining the trajectories associated with different leadership competencies in a sample of high-potential executives. This represents a novel evaluation approach to examine individual change over time during an intervention.

To further investigate the factors that are related to the development of leadership competency, we propose that leader identity will be positively associated with the developmental trajectories of leadership competencies. In this study, we conceptualize leader identity as a strength of identification with one’s leadership role. Identity evolves over time as a function of various relevant experiences, including involvement in leader development (Lord and Hall, 2005; Day et al., 2009). Leader identity is thought to be especially important in the development of leadership competencies because identity motivates an individual to engage in leadership activities and practice desired skills. We expect that leader identity forms a complex relationship across a range of leadership competencies in that it relates more strongly to the development of certain leadership competencies than others. This is due to the varying leadership schemas held by leaders, which form a part of a leader’s identity (Epitropaki et al., 2017). That said, the relationship between leader identity development and leadership skills development is complex (Miscenko et al., 2017), which mitigates any strong causal claims being made based on the current research design.

Leadership competencies have been identified as one of the factors that contribute to managerial career advancement (Claussen et al., 2014). Existing evidence suggests that organizational training and skill development opportunities are positively related to promotion (Ng et al., 2005). However, this literature lacks insights about specific leadership competencies that are crucial for promotion decisions (Collings and Mellahi, 2009; Claussen et al., 2014). The present study addresses this gap by proposing that the initial level and the change in leadership competencies will differently relate to (job) promotion following the participation in a high-potential leadership development program.

In sum, the present study contributes to the literature by advancing novel propositions about the development of leadership competencies. First, we suggest a more realistic view on the development of leadership competencies in proposing that individuals will have different initial levels of each leadership competency and these competencies will change at different rates during the leadership development program. Although these propositions are fairly straightforward and consistent with models of skills acquisition (i.e., Ackerman, 1987), this is not how leadership competencies are typically conceptualized or studied. Whereas previous literature has focused on the antecedents and the outcomes of a single competence (e.g., strategic thinking; Dragoni et al., 2011), our study advances a more complex model of competencies required for effective leadership at the executive level.

Second, we propose that leader identity may be associated with differences in the rate of competency development as a knowledge structure that supports competency acquisition. Although leader identity has been proposed as a proximal outcome of leadership development that supports the acquisition of leadership skills and competencies, it has been rarely investigated, especially among more experienced leaders participating in a high-potential leadership development program. By studying both identity and competencies, we are able to provide a fuller account of executive leadership development. Furthermore, given that more experienced executives have had greater exposure to leadership opportunities and may have engaged in leader identity development as compared with emerging leaders, there is likely to be less variability across more mature individuals in terms of how strongly they perceive themselves as leaders. Therefore, finding evidence of leader identity effects among experienced leaders poses a conservative test of the hypothesized relationships. Third, we evaluate the outcomes of leadership development by studying the promotion outcomes among the participants. Career outcomes are rarely investigated in leadership development literature, although it assumes that increases in human capital will lead to career advancement among leaders. We extend this research by proposing that a change in different leadership competencies will differently predict (job) promotion among program participants.

Overall, the present study contributes to the research and the practice of leadership development. Recent meta-analysis has demonstrated the overall effectiveness of leadership training on the outcomes of reactions to the training, learning, behavior transfer, and organizational results (Lacerenza et al., 2017). However, it is important to distinguish between leadership training and development initiatives. Leadership training tends to be focused, structured, short-term interventions in which all participants are expected to acquire the same knowledge and skills. Leadership development tends to be of a longer term, more individualized, and focused on senior leaders to expand individual and collective capacity for effective leadership (Day et al., under review). Evidence for the effectiveness of leadership development initiatives lags behind the evaluation research on leadership training. One purpose of the present research is to address this gap.

Finally, the present study contributes to leadership development practice by describing a novel way of designing and conducting leadership development evaluation studies. We demonstrate how existing organizational competency models can support tracking leader development by coaches over time using sophisticated, yet straightforward, statistical tools.

## Conceptual Background and Hypotheses Development

### Leadership Competencies

We define leadership competency as a composite of knowledge, skills, and abilities required to perform effectively in a leadership role (see McCall et al., 1988). Unlike generic leadership behaviors and skills that are relevant to leadership roles regardless of the context, leadership competency is highly dependent on the tasks and the objectives of a particular leadership role (Hollenbeck et al., 2006). For example, strategic thinking competency is needed to address a specific type of organizational problem – how to best achieve organizational growth and ensure long-term viability (Dragoni et al., 2011). Not surprisingly, researchers have developed a range of leadership competency models for specific occupations like health services (Hopkins et al., 2015), higher education, or project management (Müller and Turner, 2010); companies (e.g., 3M; Alldredge and Nilan, 2000) or domains of practice (e.g., cross-cultural competency; Caligiuri and Tarique, 2012). Many of these competency models have been linked to effective leadership in the respective roles and domains. It is also the case that leadership competencies are less amenable to training than knowledge, skills, and ability that are more straightforward and suggest adopting longer term, experience-based, developmental initiatives (Day et al., under review).

Experience – and especially its lessons (McCall et al., 1988) – has been proposed as the most potent way to develop leaders’ competencies (McCall, 2004, 2010). This is consistent with the proposition that leaders accumulate complex skills over time in a progressive, systematic fashion (Mumford et al., 2000a). For example, accumulated work experience was positively related to executives’ strategic thinking competency after controlling for individual characteristics (Dragoni et al., 2011). Other recognized ways to develop leadership competency are through training interventions, such as a formal leadership development program (Day and Dragoni, 2015), which is a more structured form of experience. Training contributes to the development of leadership competencies because it provides a set of systematic experiences that aid in acquiring new leadership-related knowledge and opportunities to practice new skills and abilities (Lacerenza et al., 2017). For example, because leadership training typically exposes participants to novel leadership concepts, it facilitates the accumulation of new leadership-related knowledge. In turn, this increased knowledge enables leaders to perform in their leadership roles more effectively (Lord and Hall, 2005).

Moreover, effective leadership training often encourages participants to practice their newly acquired skills (Lacerenza et al., 2017) through various forms of experiential learning activities, such as business simulations, role plays, and team projects (i.e., action learning). This is in line with several learning theories. The social learning theory of Bandura (1977) proposes that human behavior is learned by observation, and thus the experiences of others guide the subsequent behavior of the incumbent. Similarly, the experiential learning theory (Kolb, 1984) proposes that knowledge is created through the transformation of experience. Such competency development through experiential learning enables leaders to understand, amplify, and anticipate contexts, situations, or reactions, expanding their capacity for action and adaptation in the leadership role (Daloz Parks, 2005; Avolio et al., 2009). Empirical research also supports these claims by showing that participants undergoing leadership training report higher levels of leadership skills (Mumford et al., 2000a; Hirst et al., 2004).

Finally, leadership coaching included as part of leadership development programs is beneficial for the development of leadership competencies because coaches challenge leaders to adopt new perspectives (i.e., new knowledge) and prompt leaders to reflect how to use existing and new competencies to address work challenges (Ladegard and Gjerde, 2014). Importantly, coaching is a flexible individualized process that attends to the particular needs of the leaders and their respective organizations; thus, it allows focusing on the development of specific leadership competencies. Scholars have long recognized the importance of providing such support to developing leaders during critical experiences (Day and Dragoni, 2015). Empirical evidence suggests that leadership coaching is beneficial for acquiring new skills (Smither et al., 2003) and developing self-efficacy in applying these skills (Baron and Morin, 2010). Moreover, a combination of workshops and individual coaching had a positive effect on leadership competencies among first-line managers (Rappe and Zwick, 2007).

In sum, available theoretical and empirical evidence suggests that leadership competencies change during development initiatives. Extending these findings, we propose that leadership competencies will develop at different rates (see also Lord and Hall, 2005). Leaders will be quicker to acquire straightforward technical and social competencies that rely on simpler knowledge structures than creative problem solving and system competencies that rely on complex integrated knowledge structures (Mumford et al., 2000a, b). The development of complex integrated knowledge structures requires more time (Lord and Hall, 2005) and partly depends on the work experiences leaders are exposed to. Performing similar work activities multiple times increases the likelihood of eventual mastery, which means that leaders become more competent in performing a specific task (Dragoni et al., 2011). This is consistent with the notion that repetition is instrumental for skills development (Gagné and Medsker, 1996). For example, leaders will frequently negotiate and manage stress but will less frequently challenge the *status quo*. Therefore, it could be expected that their competency in negotiating will develop at a faster rate compared to the more complex and less frequently practiced competency of challenging *status quo*. Finally, training interventions might not address all competencies equally well. This is again because it is easier to design and facilitate learning experiences that address technical knowledge competencies.

Such differences in the rate of skill acquisition have been primarily studied in comparing junior to senior leaders. For example, Mumford et al. (2000a) investigated the acquisition of leadership skills over the course of a leader’s career. Their findings for the social judgment competency suggest that simpler skills (i.e., systems perception) developed at a faster rate among junior to mid-level leaders than more complex skills (i.e., solution fit), which developed faster among middle-level to senior leaders. However, we argue that leadership competencies will also develop at different rates even when comparing leaders at the same level (i.e., high-potential executives) because some competencies are more complex and require a longer time to develop (i.e., valuing diversity, creating commitment). In addition, executives may choose to prioritize the development and the practice of certain competencies, but not others, because they assign varying importance to their leadership competencies (Semeijn et al., 2014).

Finally, there will also be differences in the initial level of different leadership competencies due to differing work experiences accumulated by the program participants (e.g., Dragoni et al., 2011). Previous research has acknowledged that leaders entering into a leadership development program will have differing experiences, skills, and learning styles (Solansky, 2010). In addition, Day and Sin (2011) found evidence of different types of developmental trajectories of leader effectiveness. Because leader effectiveness relies on the mastery of leadership competencies, we expect that distinct leadership competencies will develop at different rates. We expect that the shape of that development will be positive and linear.

Hypothesis 1: The developmental trajectories of coach-rated leadership competencies of the high-potential executives in a leadership development program will be positive over time.

Hypothesis 2: There is a difference in the (a) starting level (i.e., intercept) and the (b) change (i.e., slope) of developmental trajectories of coach-rated leadership competencies.

### Leader Identity Development

Leader identity refers to the “sub-component of one’s identity that relates to being a leader or how one thinks of oneself as a leader” (Day and Harrison, 2007, p. 365). The identity theory (Stryker and Burke, 2000) conceptualizes identity as a collection of meanings associated with a particular social role (e.g., leadership role) an individual occupies. Leader identity is said to encompass four dimensions: (a) meaning, (b) strength, (c) integration, and (d) level (Hammond et al., 2017). In the present study, we focus on the strength dimension as it is central to how others have operationalized and measured leader identity (e.g., Day and Sin, 2011; Miscenko et al., 2017). Strength refers to the extent to which an individual identifies as a leader and the strengths of alignment with one’s leadership role. The identity dimensions of meaning, integration, and level are less important to the present research because of our focus on identity strength and the activities associated with competency practice.

The identity theory conceptualizes identity as a relatively stable and enduring entity (Miscenko and Day, 2016). Nevertheless, changes in identity can be initiated by external events, such as significant work role transitions (Ibarra, 1999) or participation in professional development activities. Specifically, it has been proposed that leadership development programs induce change in leader identity strength because an individual is exposed to new information about identity and is prompted to re-construct the currently held identity (Sveningsson and Alvesson, 2003; Hall, 2004), which will ultimately influence the strength of self-perception as a leader (i.e., leader identity; Miscenko et al., 2017).

Participation in a leadership development program may challenge the participants’ leader identity in several ways. First, receiving feedback from their superiors, peers, and subordinates (i.e., 360 assessment, feedback on action learning project) may expose strengths and weaknesses that will prompt the participants to reflect on their leadership and to adjust identity strength accordingly. Second, coaching could be especially relevant to the development of identity because its individualized approach facilitates deeper reflection and might lead to the discovery of the inconsistency between own identity and socially constructed understanding of the leadership role. This process of reflection and re-construction will influence the strength of leader identity. In a qualitative study, mentoring has been found to influence the discovery and the development of leader identity (Muir, 2014). Third, participants may be confronted with an idealized description of a leadership role (e.g., examples of prominent leaders), which motivates them to re-construct their own leader identity (Gagnon and Collinson, 2014).

Although empirical evidence is available to support these propositions, previous findings are often based on student samples. For example, students exposed to transformational leadership examples reported a significant increase in transformational leader role identity compared to a control group (Waldman et al., 2013). Studies based on managerial samples similarly indicate that a leadership development program has a positive effect on the participants’ identification with a managerial role (Rappe and Zwick, 2007).

Extending these findings, we argue that high-potential leaders will experience a positive change in the strength of their leader identity during a leadership development program. This is because the same identity development mechanisms operate across different age and experience groups (Bosma and Kunnen, 2001). Therefore, experienced leaders participating in a leadership development program are likely to encounter new information that conflicts with their currently held identity. However, in contrast to previous research that found a curvilinear, J-shaped developmental trajectory of leader identity among graduate students in a leader development program (Miscenko et al., 2017), we propose that with highly experienced executives there will not be any initial loss in the strength of leader identity. This is because their leader identity is likely to be stable given the breadth and the depth of leadership experience. For example, Kragt and Guenter (2018) found that the identity of inexperienced leaders was more affected by leadership training than that of experienced leaders.

Thus, we expect the developmental trajectory of leader identity to be linear and positive among highly experienced executives.

Hypothesis 3: The developmental trajectories of self-rated leader identity of the high-potential executives in a leadership development program will be positive over time.

### Leader Identity and Leadership Competency

Drawing from an integrative model of leader development, we propose that leader identity will be associated with the development of leadership competencies. Specifically, it has been proposed that, as a knowledge structure (i.e., deep level), leader identity supports the observable, behavioral level of leadership competencies (i.e., surface level) (Day et al., 2009; Day and Sin, 2011). Identity supports the acquisition and the integration of leadership competencies (Lord and Hall, 2005). Identity also provides a basis for motivation to further develop leadership competencies in that a strong sense of self as a leader will motivate an individual to seek out opportunities to practice and to further develop one’s leadership competencies (Day et al., 2009). This is because humans are motivated to act consistently with their self-concept (Rosenberg, 1979) or, in other words, thinking motivates doing (Fiske, 1992). In support of these propositions, previous research has found that leader identity is positively related to the acquisition of leadership skills (Miscenko et al., 2017) and the expression of leader behaviors underlines these skills (Johnson et al., 2012). Nonetheless, the current research design precludes any strong claims of the causal relationship between leader identity and leadership competency development.

Extending these previous empirical and theoretical insights, we suggest that leader identity will differentially relate to the development of leadership competencies. Specifically, we propose that leader identity will form different relationships with the developmental trajectories (i.e., initial levels and changes) in different leadership competencies. Although this argument makes an intuitive sense, there is little theoretical guidance available to explain why this might be the case. Possibly, leader identity is more strongly related to the acquisition of those competencies that are seen as representative (i.e., prototypical) to the meaning of leadership held by an individual. This is in line with the literature on implicit leadership theories, which proposes that individuals hold leadership schemas of prototypical leaders (Lord et al., 1984; Epitropaki and Martin, 2004). In addition, previous research has found that leader identity forms different relationships with leadership skills of initiating structure and consideration (Miscenko et al., 2017). Thus, we conduct exploratory analyses to examine whether leader identity is differentially associated with leadership competencies and their development.

### Leadership Competencies and Promotion

Finally, to investigate tangible outcomes of participation in a leadership development program, we examine how the initial level and the change in leadership competencies relate to (job) promotion after the program. In line with the human capital theory (Becker, 1975; Strober, 1990), developmental programs are used to enhance leaders’ competencies, which are linked to organizational performance. Thus, individuals are motivated to invest in their development because this will allow them to advance their career. There are increasingly fewer positions the higher one rises in a corporate organization, which some have labeled as tournament mobility (Rosenbaum, 1979). It is also in the best interest of organizations to invest selectively in the development of their high-potential leaders and promote those with the most developed leadership competencies (i.e., human capital) in the hopes of achieving better performance. For example, research has demonstrated that there is a link between executive level leadership and organizational performance (e.g., Day and Lord, 1988).

These propositions have been supported empirically. Early research demonstrated that on-the-job training is positively correlated with promotion (Sheridan et al., 1997). In a meta-analytical review of predictors of career success, Ng et al. (2005) found that organizational training and skill development opportunities are positively correlated with promotion. More specific to the leadership domain, an increase in human capital (i.e., expertise) was found to improve the promotion odds for middle- and senior-management positions (Claussen et al., 2014).

Extending these findings, we propose that initial level and change in different leadership competencies will differently relate to promotion. This is because those making promotion decisions may consider some leadership competencies as more important for higher-level positions (see Breaugh, 2011). Thus, even if a leader has a relatively high initial level of a given leadership competency and experiences improvement in that competency through participating in a leadership development program, he or she may still not be promoted if this competency is not considered as important for a higher-level leadership position. Supporting this proposition, Semeijn et al. (2014) found that supervisor-rated results-oriented competencies were positively related to perceived managerial effectiveness, but relationship-oriented competencies were not.

Hypothesis 4: Initial level (i.e., intercept) and change (i.e., slope) of leadership competencies will predict the promotion of high-potential executives following participation in a leadership development program.

## Materials and Methods

### Participants and Procedure

The Human Research Ethics Committee at the University of Western Australia approved the study. All participants were provided with a study information sheet and have signed an informed consent form. The sample consisted of executives nominated by their respective businesses as high-potential senior leaders, employed by a large Australian conglomerate (200,000 + employees), to participate in a corporate leadership development program. Each year, a cohort of 14–19 participants engaged in a 5-month program. Members of the HR staff at the parent company headquarters screened all nominees for high potential suitability. The program participants that passed the headquarters’ screening were invited to an initial 2-day orientation. The orientation included an overview of the program’s goals and action learning approach. One of the authors provided an overview of the research component and collected informed consent from the participants.

The program participants met in person for 2 days, approximately every 5–6 weeks, at different company locations. The activities varied by session but included self-assessments and feedback on individual differences such as personality, decision-making, risk tolerance, and goal orientation. The participants engaged in leadership simulations (e.g., Looking Glass Experience; see Seltzer, 1989) and skill building activities, such as providing feedback, active listening, and other communication skills. The core of the development intervention was a team-based action-learning project, supported by individual and group coaching. Each action learning team worked on a project offered by a corporate sponsor. The results were presented to the parent company’s corporate senior leadership team, including the chief executive officer. It was emphasized that each team should focus not only on delivering an excellent project (i.e., action) but also documenting how that project enhanced their development individually and collectively (i.e., learning). The research team did not have access to any of the other assessments or deliverables other than the self-rated leader identity scale and coaches’ ratings.

The data were collected over 5 years with a total sample size of 80. All ratings were collected electronically from participants and coaches the week following a multi-day engagement session that occurred at various points throughout the broader program. Measures were administered at four different time points (T0–T3) during the program, approximately 4–5 weeks apart.

About a quarter (26.6%) of the participants were female, and the average age was 38.6 years. All participants held a formal leadership role prior to and during the program. Two-thirds (66.3%) of the participants had a senior management position role at the time of the program (e.g., manager of technical services, financial services business manager), 22.5% were in a managerial or senior professional role (e.g., business analyst), and 7.5% were in a general management role (e.g., business category manager, regional manager). The response rate among the program participants varied between 82.5 and 90.0% (86.3% on average).

The coaches provided executive coaching from the same external vendor across cohorts at the individual level and the team level during the multi-day engagement sessions and throughout the program by phone. Ten coaches were engaged with the program, providing both individual and group coaching sessions. The number of participants per coach across five program years varied between 3 and 21 (average = 5.1). Six coaches participated in the program just once, one coach twice, one coach three times, one coach four times, and finally, one coach participated in all five program years. Three of the coaches were female.

### Measures

Leader identity was self-reported by the study participants using an established three-item measure (Hiller, 2005; Day and Sin, 2011). The participants were asked to rate on a five-point scale (1 = *never or rarely to 5* = *always*) how consistently they describe and see themselves as leaders. The items were “I am a leader,” “I see myself as a leader,” and “Being a leader is important to me.” The internal consistency (alpha) estimates for the leader identity measure were generally acceptable (T0 = 0.74, T1 = 0.66, T2 = 0.76, and T3 = 0.73).

Eight distinct leadership competencies were measured *via* a single item, each derived from a company’s leadership competency framework. The framework was developed by the company prior and independent of the current study. The coaches rated these competencies on four different occasions during the leadership program. Although single-item measures are not ideal because of potential reliability concerns, longer assessments were not allowed especially that the surveys were completed across multiple measurement waves. The competencies measured were challenging the *status quo* (SQ), valuing diversity (VD), promoting employee voice (PEV), creating commitment (CC), negotiating (N), managing stress (MS), articulating complex ideas (ART), and adapting to change (AD). The items used to measure each competency are presented in Appendix.

We extracted the information about promotion (outcome) from company records 1 year after the last cohort of high-potential executives completed the program. Promotion was coded 1 if a participant changed the role within the same hierarchical level or was promoted to the next level position. Promotion was coded 0 if a participant stayed in the same role, including those who left the organization since the conclusion of the program. Overall, 56.3% of the program participants were promoted subsequent to program participation.

#### Control Variables

Because 10 different coaches provided ratings of leadership competencies, we controlled for individual coaches in all analyses. We created 10 binary variables (i.e., 0 and 1) – one for each coach – and included these variables in all analyses with first coach serving as a baseline. We also controlled for a participant’s gender as prior evidence suggests that men and women may differ in their leader self-perception (Day and Sin, 2011). For example, females have been shown to rate themselves lower across a range of leadership competencies compared with men (Mayo et al., 2012). Finally, when predicting promotion outcomes, we controlled for the year in which the participants took part in the leadership development program because those who took part in the earlier program may have had greater chances of promotion due to longer post-program tenure. Each program year was coded as a binary variable (i.e., 0 and 1), with the first program year serving as a baseline in the analyses.

### Analytical Strategy

Data were conceptualized at two levels of analyses – between individuals (level 2, i.e., promotion outcome, gender, coach) and within an individual (level 1, i.e., leader identity, leadership competencies) and the hypotheses involved relationships between variables at the same and at different levels of analysis. Considering this data structure, hierarchical linear modeling (HLM; Bryk and Raudenbush, 1992) was used for analysis since it allows testing for interactions between variables at different levels of analysis and accounts for different sources of variance (Mathieu et al., 2012). The hypotheses were tested using the non-linear and the linear mixed effects program for R (Pinheiro and Bates, 2006), following the analytical procedures outlined by Bliese and Ployhart (2002) in R version 3.0.1. Within-individual predictors (i.e., leader identity) were centered on the person mean. Centering week-level variables at the person mean implies that the random effect of these time-varying covariates will be based on within-person variation (Hoffman and Stawski, 2009), which is appropriate given the aims of the present study. Deviance statistics (−2 log likelihood) was used for comparing models in terms of fit. The missing values were omitted in the analyses. The specific analyses conducted are explained in the “Results” section.

## Results

The descriptive statistics and the intercorrelations for all study variables are included in Supplementary Material. The respective means of all leadership competencies and leader identity increase over time, indicating a positive, linear development. This, in itself, is insufficient evidence for the presence of growth in competencies; hence, we conduct analyses of developmental trajectories for each of the respective leadership competencies.

### Leadership Competencies

Although the aim of the present study was to investigate the differences in development of leadership competencies over time, we conducted exploratory factor analyses with the eight measured leadership competencies to establish the underlying factor structure. We chose principal axis factoring as the extraction method (Fabrigar et al., 1999). The factors were free to vary based on the traditional Eigenvalue cutoff of 1.0. Across four measurement points, all eight leadership competencies loaded on one factor that explained more than 50% of the variance in the items (T0, 51.67%; T1, 54.5%; T2, 54.1%; and T3, 63.3%). The factor loadings were acceptable (T0:0.58–0.75, T1:0.58–0.80, T2:0.53–0.83, and T3:0.69–0.85). These results suggest that the eight leadership competencies describe a single underlying construct, yet we also note that the inter-correlations between competencies are not so high as to suggest that they are measuring the same construct, which is suitable for our study purposes as we investigate the differences between competencies.

Table 1 reports the results of the model estimation, including coefficients, results of variance decomposition, and model fit. To test hypothesis 1, the analyses were conducted in three steps. First, we estimated an unconditional means model (intercept only, model 0) to determine the intraclass correlation coefficient, which indicates how much of the total variance in leadership competencies varies between versus within individuals. Between 35.8 (promoting employee voice) and 50.5 (managing stress) percent of variance in different leadership competencies was attributable to within-person variation. These findings suggest that leadership competencies considerably fluctuated over time, thereby suggesting that multilevel analysis is an appropriate data analysis strategy.

**TABLE 1:** Modeling the development trajectory of leadership competencies (hypothesis 1).

	Leadership competency							
	SQ	VD	PEV	CC	N	MS	ART	AD
**Model 0: intercept only**								
ICC	0.413	0.452	0.358	0.440	0.424	0.505	0.466	0.459
−2LL (*df* = 3)	727.5	726.1	811.9	739.6	701.6	717.7	715.0	686.7
**Model 1a: fixed slope**								
Intercept	2.78*	2.90*	2.95*	2.89*	2.93*	3.25*	2.78*	2.83*
Linear slope	0.269*	0.291*	0.297*	0.213*	0.231*	0.205*	0.256*	0.305*
−2LL (*df* = 4)	640.2	617.1	734.9	696.4	635.9	664.1	630.0	567.5
**Model 1b: varying slope**								
Intercept	2.78*	2.90*	2.95*	2.89*	2.93*	3.25*	2.76*	2.83*
Linear slope	0.269*	0.290*	0.296*	0.213*	0.230*	0.204*	0.256*	0.306*
−2LL (*df* = 6)	636.8	615.8	725.8	690.8	631.8	648.6	629.8	564.5
Δ−2LL (Δ*df* = 2)	3.4	1.3	9.1*	5.5	4.1	15.5*	0.2	3.0
**Model 2: varying slope and control variables^*a*^**								
Intercept	3.37*	3.67*	3.43*	4.03*	3.68*	4.07*	3.19*	3.82*
Linear slope	0.269*	0.291*	0.296*	0.213*	0.229*	0.205*	0.255*	0.303*
Gender	−0.072	−0.548	0.324*	0.098	0.004	−0.217	−0.022	−0.110
−2LL (*df* = 16)	581.5	570.0	580.0	639.5	563.9	584.0	588.9	484.5

*SQ*, challenging the status quo; VD, valuing diversity; PEV, promoting employee voice; CC, creating commitment; N, negotiating; MS, managing stress; ART, articulating complex ideas; AD, adapting to change; LL, log likelihood; df, degrees of freedom. ^*a*^Model 2 analyses included control variables for coaches (dummy coded). *Significant at p < 0.05.

Next, we estimated two models with a time variable to account for a possible linear trend in leadership competencies. In model 1a, the slope for time was fixed, but in model 1b the slopes were allowed to vary across time. The two models were compared in terms of fit. For most leadership competencies (except promoting employee voice and managing stress), the model with time-varying slope was not significantly better than the model with a fixed slope. However, in line with previous research indicating that cross-level interactions should be tested regardless of the significance of slope variance (Bliese and Britt, 2001; LaHuis and Ferguson, 2009), we retain the time-varying slope models for further analyses. We also tested for quadratic slope in leadership competencies; however, quadratic slope was insignificant for almost all leadership competencies (except promoting employee voice)^1^. Thus, a model with time-varying linear slope was found as a better choice when describing the developmental trajectory of most leadership competencies in the present study. Level 1 models also controlled for autocorrelation.

Finally, the next modeling step was the inclusion of control variables (model 2). Supporting hypothesis 1, the findings suggest that, while controlling for gender and coaches, all of the leadership competencies developed along a linear, positive trajectory with the following linear slope estimates: challenging the *status quo*, β = 0.27, *p* < 0.001; valuing diversity, β = 0.29, *p* < 0.001; promoting employee voice, β = 0.30, *p* < 0.001; creating commitment, β = 0.21, *p* < 0.001; negotiating, β = 0.23, *p* < 0.001; managing stress, β = 0.20, *p* < 0.001; articulating complex ideas, β = 0.26, *p* < 0.001; and adapting to change: β = 0.31, *p* < 0.001. Thus, hypothesis 1 is supported.

Hypotheses 2a and 2b proposed that despite the similar developmental trajectory of leadership competencies, there will be differences in their starting level (i.e., intercept) and the rate of change (i.e., slope). To test this hypothesis, we first extracted the individual participants’ intercept and slope coefficients for each leadership competency. These coefficients were obtained from model 2 and thus included the effects of control variables (coach and gender). Next, we conducted ANOVA analyses to establish whether the individuals’ intercepts and slopes are different between leadership competencies. We used the robust Welch test (Kohr and Games, 1974) due to heterogeneous variance (Levene’s test for intercepts: 6.68, *p* < 0.001; slopes: 32.51, *p* < 0.001). The results suggest that both intercepts [*F*(7, 270) = 50.71, *p* < 0.001] and slopes [*F*(7, 238) = 15.07, *p* < 0.001] were significantly different across leadership competencies. The *post hoc* analyses (multiple comparisons) suggested that majority of the intercept coefficients were significantly different when the competencies were compared one to one. Somewhat an exception was adapting to change competency, which was only significantly different from three other competencies (challenging the *status quo*, promoting employee voice, and articulating complex ideas). Furthermore, the *post hoc* analyses showed that most competencies’ slope coefficients were also significantly different. A notable exception was the articulating complex ideas competency slope that did not significantly differ from any other competency slope. Overall, both hypotheses 2a and 2b are supported.

### Leader Identity and Competencies

To test hypothesis 3 (the developmental trajectory of leader identity), we followed the same modeling steps as with estimating the developmental trajectory of leadership competencies. The unconditional means model (intercept only, model 0) indicated that 30.0% percent of variance in leader identity was attributable to within-person variation (see Table 2). The model with time-varying slope (model 1b) was not significantly better than the fixed-slope model (model 1a). However, as discussed above, we retained the time-varying model for future analyses. The quadratic slope coefficient was not significant. In model 2, we included gender as a control variable. The results of model 2 suggest that, similar to leadership competencies, leader identity developed along a linear, positive trajectory (linear slope β = 0.186, *p* < 0.001). Thus, hypothesis 3 is supported.

**TABLE 2:** Modeling the development trajectory of leadership competencies (hypothesis 3).

	Leader identity
**Model 0: intercept only**	
ICC	0.300
−2LL (*df* = 3)	414.2
**Model 1a: fixed slope**	
Intercept	3.95*
Linear slope	0.183*
−2LL (*df* = 4)	329.4
**Model 1b: varying slope**	
Intercept	3.94*
Linear slope	0.186*
−2LL (*df* = 6)	324.3
Δ−2LL (Δ*df* = 2)	5.1
**Model 2: varying slope and control variables**	
Intercept	3.98*
Linear slope	0.186*
Gender	−0.139
−2LL (*df* = 16)	323.0

LL, log likelihood; df, degrees of freedom. *Significant at p < 0.05.

Next, we investigated the relationships between leader identity and different leadership competencies. We included leader identity as a time-varying predictor in the models retained from previous HLM analyses. Table 3 reports the results of the model estimation, including coefficients and model fit. The findings suggest that leader identity is significantly and positively related to three leadership competencies (challenging the *status quo*: β = 0.227, *p* < 0.05, valuing diversity: β = 0.293, *p* < 0.01, and creating commitment: β = 0.190, *p* < 0.05). The results suggest that leader identity is positively related to the development of three of eight (approximately 38%) of the leadership competencies over time.

**TABLE 3:** Modeling the effect of leader identity on leadership competency trajectory.

	Leadership competency							
	SQ	VD	PEV	CC	N	MS	ART	AD
Intercept	3.52*	3.79*	3.45*	3.29*	3.65*	4.01*	3.28*	3.92*
Linear slope	0.226*	0.237*	0.297*	0.167*	0.249*	0.234*	0.252*	0.283*
Gender	−0.044	0.122	0.364*	0.110	−0.061	−0.265*	−0.037	−0.107
Leader identity	0.227*	0.293*	−0.097	0.190*	−0.016	−0.163	0.012	0.084
−2LL (*df* = 17)	476.4	480.6	580.3	548.0	471.2	506.1	487.5	389.3

SQ, challenging the status quo; VD, valuing diversity; PEV, promoting employee voice; CC, creating commitment; N, negotiating; MS, managing stress; ART, articulating complex ideas; AD, adapting to change; LL, log likelihood; df, degrees of freedom. All analyses included control variables for coaches (dummy-coded). *Significant at p < 0.05.

### Promotion

Hypothesis 4 proposed that the initial level (i.e., intercept) and the change (i.e., slope) of leadership competencies will differently relate to (job) promotion after the leadership development program. To test this hypothesis, we conducted analyses using binary logistic regression in SPSS. The intercept and slope coefficients for each leadership competency were obtained from model 2 (previous HLM analyses) and thus included the effects of control variables (coach and gender). We also controlled for program year in these analyses because those who took part in the earlier program may have had greater chances for promotion due to longer post-program tenure. Table 4 reports the results of the analyses, including coefficients and model fit. The findings suggest that initial level and change in some leadership competencies were related to (job) promotion post-program. Specifically, initial level/change in valuing diversity leadership competency was significantly and negatively related to promotion (intercept β = −6.35, *p* < 0.01; slope β = −36.8, *p* < 0.01). Similarly, level/change in negotiating was significantly and negatively related to promotion (intercept β = −28.2, *p* < 0.05; slope β = −36.8, *p* < 0.05). Change, but not initial level, in managing stress was positively and significantly related to promotion (β = 7.65, *p* < 0.05). All other leadership competencies were unrelated to promotion. Thus, hypothesis 4 is partially supported.

**TABLE 4:** Modeling the effect of competency trajectory on promotion (hypothesis 4).

	Leadership competency							
	SQ	VD	PEV	CC	N	MS	ART	AD
Constant	2.09	34.4*	10.3	5.66	133.0*	−6.08	−13.6	−45.5
Year 2011	0.294	−0.285	0.215	0.170	0.074	0.348	0.630	0.293
Year 2012	0.245	0.347	0.249	0.275	0.177	0.270	0.218	0.294
Year 2013	0.650	0.267	0.587	0.544	0.293	1.40	0.736	0.689
Year 2014	0.117	−0.377	0.196	0.142	0.016	0.198	0.185	0.141
Intercept coefficient	−0.445	−6.35*	−0.2.36	−1.26	−28.2*	1.12	0.782	7.98
Linear slope coefficient	−1.82	−36.8*	−7.19	−2.05	−127.5*	7.65*	43.3	49.9
Pseudo-*R*^2^	0.022	0.210	0.036	0.064	0.108	0.137	0.044	0.027

SQ, challenging the status quo; VD, valuing diversity; PEV, promoting employee voice; CC, creating commitment; N, negotiating; MS, managing stress; ART, articulating complex ideas; AD, adapting to change. *Significant at p < 0.05.

## Discussion

Leadership competence is an essential foundation for effective leadership, especially at the executive level. Extending the leadership literature that has typically evaluated the development of a single leadership competency as a function of a leader’s experience (e.g., Dragoni et al., 2011), the present study proposed that high-potential executives rely on a complex set of leadership competencies in performing their leadership role. We investigated how these distinct leadership competencies develop over time among participants in a highly selective corporate leadership development program as well as the antecedents and the outcomes of their development. Our findings support the proposition that there are differences in the rate of development of various leadership competencies. We also find that leader identity relates to the development of some of these leadership competencies and that (job) promotion can be predicted by a subset of these competencies. We discuss these findings and their theoretical and practical implications in the following sections.

### Leadership Competencies

The findings of the present study demonstrate that leadership competencies develop along a generally positive, linear developmental trajectory during a 5-month-long leadership development program. This is consistent with previous empirical evidence suggesting that leadership skills and leadership effectiveness develop in the overall positive pattern during the leadership development interventions (Day and Sin, 2011; Miscenko et al., 2017). More importantly, we find that the initial level and the rate of change differ across the different leadership competencies. We proposed that this is because leadership competencies are underlined by different knowledge structures, which differ in their complexity. More complex knowledge structures require longer timescales to develop (Lord and Hall, 2005). It is also likely that executive leaders use some of the competencies more frequently; specifically, those that are required for everyday job tasks are most likely to benefit from more frequent practice. The repetitive use of competencies will mean that these develop at a faster pace, especially in combination with a leadership development program which offers additional opportunities for practicing knowledge, skills, and abilities that underlie key leadership competencies.

More specifically, looking at the results for each leadership competency analyzed in the present study (see Table 1), we find that (after controlling for coach and participant gender) the participants had the highest initial level of the managing stress competency and the lowest initial level on the competency of articulating complex ideas. Contrasting these two competencies, articulating complex ideas potentially relies on a more complex underlying knowledge structure than managing stress. For example, Lord and Hall (2005) proposed that leaders develop principled-level knowledge that allows them to define problems and environments in terms of underlying principles rather than surface-level features. Clearly articulating complex ideas will require a deeper, expert-level understanding that develops with experience and deliberate practice (Ericsson and Charness, 1994). On the other hand, research suggests that managers consistently report a higher level of stress (Skakon et al., 2011); thus, the managing stress competency is better developed due to a more frequent need to use it. In addition, we find that the managing stress competency developed at the slowest rate during the leadership development program. This may be because the senior leaders in our sample already achieved a higher level of expertise in that competency by virtue of their executive-level positions and high-potential leadership status. Finally, we find that the competency of adapting to change developed at the fastest rate. We suspect that this is because participation in the leadership development program in itself represented a disruptive experience that required participants to become more open to changes. Typical of such interventions, the participants take on the additional developmental responsibilities (e.g., action learning project) on top of their everyday works. This is consistent with previous findings suggesting that the participants in leadership development programs experience a considerable uncertainty (Nicholson and Carroll, 2013).

Overall, our findings are robust in that these align with previous conceptual literature (Lord and Hall, 2005), limited empirical findings of the differences in skills among novice and expert leaders (e.g., Mumford et al., 2000a), and rely on other ratings of leadership competencies and not self-report. Furthering previous research, our findings suggest a need for a more nuanced approach in the study of leadership competencies, paying closer attention to complex relationships in the development of individual competencies. It could be the case that due to the complexity of development at the executive level, the participants prioritize the development of some competencies over others. One factor that could affect this prioritization is the leadership schema contained in one’s leader identity. We explore this possibility in the next section.

### Leader Identity and Leadership Competencies

There is an increasing recognition among leadership researchers that identity processes play an important role in motivating and supporting the personal growth of leaders (Day and Harrison, 2007; Day et al., 2009; Day and Dragoni, 2015). One of the goals of this research was to demonstrate the relevance of leader identity even among senior executives for whom thinking of oneself as a leader would already be expected (to varying degrees). Because high-potential executives are likely to have internalized a leader identity to a larger extent than emerging leaders, this study represents a conservative yet important test of the underlying relationships between identity processes and leader development. Indeed the present results showed that whereas leader identity ratings demonstrated a positive trajectory over the course of the program, there was a notable ceiling effect on the ratings by the program’s end (average of 4.6 out of 5.0 possible). Furthermore, we did not find evidence of a curvilinear trajectory in leader identity, as observed in a previous research with student samples (e.g., Miscenko et al., 2017), suggesting that whereas leader identity is relevant to the development of executives, the trajectories of that development differ from more novice (i.e., student) leaders. Thus, we extend the research in the leadership development domain and demonstrate that leader identity remains highly relevant for the development of more experienced leaders as it is associated with the development of leadership competencies.

The present study finds that adopting a leader identity (self-rated) is associated with the development of some distinct leadership competencies (coach-rated) over the course of a 5-month executive development program. Specifically, we find that leader identity is positively related to developmental trajectories of three leadership competencies: challenging the *status quo*, valuing diversity, and creating commitment. We proposed that leader identity will more strongly relate to the acquisition of those competencies that are seen as more representative (i.e., prototypical) of an effective leader. Possibly, the three leadership competencies that were associated with leader identity represent part of the core leadership schema at the studied organization because leader identity is grounded in a specific social role (i.e., head of the department at company X), which is associated with certain social expectations of the incumbent’s behavior (Stryker and Burke, 2000). Interestingly, all eight leadership competencies included in the present study were represented in the company’s leadership competency framework. This suggests that, although all eight competencies are explicitly communicated by the organization as important, only some are implicitly tied to the identity of an effective organizational leader. It is also possible that the three competencies are more ingrained in the individual’s personal (as opposed to collective) leadership schema. In other words, executives see valuing diversity, creating commitment, and challenging the *status quo* as embedded in their personal meaning of effective leadership. Future research could further investigate the differences in implicit and explicit leadership prototypes at both the individual and the organizational levels and how these affect leadership development.

Another potential explanation for why leader identity is differently related to competency development is that participation in the intervention in itself challenges the participant’s view of competencies required to be an “effective” leader. This is consistent with our discussion on the impact of leadership development initiatives on the meaning of leader identity. Furthermore, it is also possible that the relationship between leader identity and competency development is reciprocal and potentially mutually reinforcing. This is consistent with the notion of identity-development spirals (Day et al., 2009), which suggests that leader identity motivates individuals to engage with leadership, resulting in the development of leadership competencies. In turn, participating in leadership experiences and acting like a leader strengthens leader identity (cf. Miscenko et al., 2017). This is consistent with the self-perception theory (Bem, 1972), which proposes that individuals draw inferences about their identity from perceptions of their own behavior. We speculate that leadership competencies that are seen as more representative (i.e., prototypical) of an effective leader would have a stronger impact on the self-identity as a leader. Unfortunately, the limited sample size in the current study precludes us from testing these reciprocal effects, but we encourage future researchers to utilize advanced modeling techniques (e.g., latent change score analysis) and larger sample sizes to investigate these ideas.

In a review of the leadership development literature, Day and Dragoni (2015) proposed leader identity as a relevant self-view (along with leadership self-efficacy and self-awareness) that, in combination with leadership competencies, offers reasonable insights into proximal leader development. The results from the present research support this perspective. Furthermore, by demonstrating that the time required to develop leaders will depend on specific competencies trained, we highlight the importance of examining time in leadership. This is in line with conceptual calls to devote greater attention to temporal issues in leadership research (Bluedorn and Jaussi, 2008; Shamir, 2011). We encourage future research to incorporate time as an important consideration in leader and leadership development. What is needed is long-term longitudinal research on more distal outcomes such as changes in dynamic skills and abstractions as well as deep-level meaning-making structures and processes. Such research – although quite difficult given the time scales involved – is needed to provide a more complete picture of the lifespan of the leader development process.

### Career Outcomes

Professional development plays an important role in advancing one’s career and securing higher-level positions. We find that the initial level and change in some, but not all, leadership competencies are related to promotion subsequent to participation in the organization’s leadership development program. Possibly this is because decision-makers may view some leadership competencies as more important for the higher-level positions (Breaugh, 2011). Surprisingly, we find that the initial level and the change in two leadership competencies (i.e., valuing diversity and negotiating) were significantly and negatively related to job promotion. In some respects, this is perplexing and perhaps even troubling. There are no unequivocal explanations for this finding, but we speculate that valuing diversity might be more of an espoused rather than enacted shared value in this organization. Executives who articulate or otherwise manifest high levels of valuing diversity might (and we must again emphasize that this is speculative) be perceived as too zealous in pursuing social justice ends at the expense of focusing on shareholder value. Interestingly, we also found that leader identity is associated with the development of the valuing diversity competency. Potentially, this supports the point we made earlier that the valuing diversity competency is associated with an individual’s personal leadership schema, whereas at the organizational level is it an espoused (rather than enacted) value. In addition, our *post hoc* analyses indicate that neither the initial level nor the change in leader identity was related to job promotion in the current study.

Furthermore, if leaders have a high level of competency on negotiating, they might be perceived as overly manipulative at the expense of adopting more of a corporate mindset (i.e., negotiating better terms for the business at the expense of the overall conglomerate organization). These negative effects on promotion are likely to be somewhat dependent on other competencies or characteristics (e.g., political skill). For example, political skill was found to moderate the relationship between use of impression management tactics and supervisor ratings of performance (Harris et al., 2007); however, leader effectiveness was lower when they relied on interpersonal influence (Douglas and Ammeter, 2004). Finally, competency change, but not initial level, in managing stress leadership was positively related to promotion. Higher-level leadership positions are often associated with increased responsibility and pressure to perform; hence, it is not surprising that leaders who improve their ability to handle stress effectively have higher chances of promotion. It is interesting that we found that the managing stress competency had the highest initial level, but the slowest rate of change. Perhaps this indicates that developing this competency beyond a certain (albeit high) level is hard and, as such, having demonstrated a better progress in developing resiliency is beneficial for securing a promotion.

Overall, the present study adds to the limited literature investigating how distinct leadership skills and competencies are related to career advancement (Claussen et al., 2014). More importantly and uniquely, we show that changes in some leadership competencies (associated with participation in a leadership development program) are related to job promotion. Our findings are perplexing, yet they concur with previous research findings that effective managers differ in their actions from those managers who are promoted (Luthans, 1988). We also note that the present study investigated only a limited number of leadership competencies. Specifically, none of the competencies described delegation or building a shared leadership capacity (Day et al., 2004), which might be important for the career advancement of senior leaders. Future research should examine how these and other leadership competencies relate to job promotion and other career outcomes of leaders.

### Practical Implications

The present findings have potentially important practical implications for organizational leadership development. First, in line with calls for a more rigorous evaluation of leadership development initiatives, we demonstrate how a trajectory modeling approach can be used to evaluate the development of leadership competencies and cognitions over time. A longitudinal approach to evaluating leadership development offers a number of advantages in terms of understanding change during the actual program intervention. Most evaluation efforts only examine pre- and post-intervention changes – if at all. Given that the present intervention spanned 5 months, it is reasonable to assume that change might occur during the intervention, which was confirmed in our analyses across all eight competencies.

Second and relatedly, we demonstrate that the time required to develop leaders will depend on the specific competencies being developed. This suggests a need for a more tailored approach to leadership development. For example, our findings suggest that distinct leadership competencies develop at different rates, which is likely due to differences in the use of these competencies and underlying knowledge structures. Some of the more complex competencies (i.e., creating commitment, challenging the *status quo*) are vital to organizational success in the changing business landscape but are not used as often as other less complex competencies (i.e., managing stress). Therefore, leader development initiatives should have a greater focus on and allow a longer time for the development of more complex competencies.

Third, we suggest that leader development should consider individual and organizational developmental needs (see Day, 2000). Our findings also suggest that individual differences, such as leader identity, play an important role in facilitating the development of certain leadership competencies. This indicates that leadership self-views should be considered when designing developmental interventions. Potentially, this is even more important at the senior-leadership level because complex leadership competencies must be supported by strong underlying cognitive structures to facilitate their development (Lord and Hall, 2005). Furthermore, we find that individuals differ in the initial level and the rate of change in the leadership competencies. Again, this suggests the need for tailored development and warns against the common one-size-fits-all approach. As our study demonstrates, one way in which leadership development could be customized to individual needs within a broader program is coaching. Coaches are able to address the challenges faced by individual leaders and guide their development along a unique trajectory.

Furthermore, organizational needs should determine leadership competencies that would be targeted by developmental initiatives (Lacerenza et al., 2017). Although competency frameworks have been criticized by some scientist–practitioners (Hollenbeck et al., 2006), the criticism mostly applies to the attempts to determine universal leadership competencies that apply across all organizations and industries. However, as our study also demonstrates, leadership competency frameworks can be useful in determining leadership competencies for a single organization (see also Alldredge and Nilan, 2000) and guiding the design of leadership development programs. These frameworks are readily available at many organizations and thus can be easily adapted for evaluation. Overall, the present study suggests that leader development should be tailored as much as possible to individual participants by considering their unique differences in cognition and competencies and to the organization by considering the requirements of specific positions (e.g., Claussen et al., 2014).

Finally, the design and the methodology of the present study offer some direction for scientists–practitioners consulting psychologists, who wish to undertake a more rigorous evaluation of leadership development initiatives. A particular strength of this research is the use of coaches’ ratings of leadership competencies instead of self-reports by the program participants. Involving coaches in program evaluation presents advantages in gathering better informed and objective ratings, reflective of participant’s development, and also reducing the burden of over-surveying the participants. We also demonstrate how a sophisticated, yet easy-to-apply, analytical technique (and freely available software) can be adapted for program evaluation. In addition, the technique can be used to produce individualized feedback to the program participants as it allows extracting personal developmental trajectories and growth coefficients. This could be valuable in guiding personalized leadership development.

### Limitations

The present study is not without limitations. First, we note the relatively modest sample size (*N* = 80), which precluded us from adopting more sophisticated modeling efforts (e.g., latent change score analyses or growth mixture modeling) to analyze the longitudinal data. However, the longitudinal data on high-potential leaders, especially those participating in leadership development programs, are difficult to obtain. This is especially evident in the leader development literature that predominantly relies on student samples. Therefore, our study offers a rare investigation into the developmental needs of “real-world” senior leaders. Second, although the non-experimental nature of our research design precludes us from drawing any strong causal inferences, the present findings are consistent with a previous conceptual work (Day and Harrison, 2007) and, at the minimum, suggest that leadership competencies and leader identity contribute to leader development processes among senior leaders. Third, some limitations are associated with the measures used in the present study. Leadership competencies were assessed *via* a single item, which potentially raises reliability concerns. However, as discussed above, this was the only feasible approach to data collection given the longitudinal design and the requirements of the organization. In addition, unlike much of previous research, we used other (i.e., coaches’) ratings of leadership competencies. Leader identity was measured using an established scale (Hiller, 2005), which exhibited some range restrictions in the present sample of experienced leaders. Despite this apparent range restriction, the effects of leader identity on the developmental trajectories were still evident across three of the leadership competencies.

In summary, we believe that this study adds to existing literature in several important ways. First, we extend the research on identity and leadership to more experienced leaders and find that leader identity plays an important, yet complex, role in the development of leadership competencies, that is, leader identity relates more strongly to some leadership competencies than others. Additional research is needed to ensure that such effects are not spurious, but it opens the possibility for additional theorizing as to which particular leadership domains (i.e., competencies) are most likely to be shaped through leader identity processes. Second, we demonstrate the value of examining differences in the developmental processes of distinct leadership competencies instead of treating these in a univariate fashion. This is especially important since we find that some of the leadership competencies are related to job promotion among the study participants. Overall, the findings reinforce an important point and that is people start at different competency levels and change in different ways in the developmental journeys. This is also true for those aspiring to lead at the very highest organizational levels.

## Data Availability Statement

The datasets generated for this study are available on request to the corresponding author.

## Ethics Statement

The studies involving human participants were reviewed and approved by the University of Western Australia Human Ethics Office, approval number RA/4/1/4031. The patients/participants provided their written informed consent to participate in this study.

## Author Contributions

Both authors listed have made a substantial, direct and intellectual contribution to the work, and approved it for publication.

## Conflict of Interest

The authors declare that the research was conducted in the absence of any commercial or financial relationships that could be construed as a potential conflict of interest.

## Footnotes

1 The quadratic slope coefficient for promoting employee voice leadership competency was negative and significant (β = −0.076, *p* < 0.05) in the model that only included intercept and slopes. However, when control variables and leader identity were included in model 2, the quadratic slope coefficient was insignificant (β = −0.064, ns). Therefore, the model with a linear slope only was reported and retained for further analyses to ease the model comparison and the interpretation.

## Appendix

**TABLE 5:** Items used to measure distinct leadership competencies.

Competency	Item
Challenging *status quo*	(The individual) can depart from accepted group norms of thinking and behaving when necessary
Valuing diversity	(The individual) sees the value in others’ unique differences
Promoting employee voice	(The individual) encourages direct and open discussion about important issues
Creating commitment	(The individual) is able to pull people together around a common goal
Negotiating	(The individual) accurately senses when to give and take when negotiating
Managing stress	(The individual) is able to stay calm and perform under pressure
Articulating complex ideas	(The individual) clearly articulates even the most complex concepts
Adapting to change	(The individual) adapts to changing conditions
