# Supplementary material for: Predicting Leadership Competency Development and Promotion Among High-Potential Executives: The Role of Leader Identity
Source: Front Psychol. 2020 Aug 5;11:1816. doi: 10.3389/fpsyg.2020.01816 (PMC7419574; doi:10.3389/fpsyg.2020.01816)
Supplement: Supplementary file 1 [file Data_Sheet_1.docx]

# Supplement

Supplementary Table 1: Correlations with control variables

|  | Gender | C#1 | C#2 | C#3 | C#4 | C#5 | C#6 | C#7 | C#8 | C#9 | C#10 |
| --- | --- | --- | --- | --- | --- | --- | --- | --- | --- | --- | --- |
| Leader identity |  |  |  |  |  |  |  |  |  |  |  |
| T0 | -.18 | .06 | .15 | .12 | -.09 | -.03 | .03 | -.01 | .07 | -.16 | -.13 |
| T1 | -.20 | .14 | .06 | .14 | -.04 | -.11 | -.12 | .08 | -.02 | -.12 | .13 |
| T2 | -.01 | .08 | .17 | .15 | -.17 | -.05 | -.07 | .01 | .08 | -.03 | -.07 |
| T3 | -.19 | .07 | .20 | .16 | -.13 | .02 | .14 | -.11 | .08 | -.06 | -.12 |
| Challenging the status quo |  |  |  |  |  |  |  |  |  |  |  |
| T0 | -.00 | .20 | .11 | .21 | .18 | **-.39** | -.09 | .20 | -.09 | .18 | -.26 |
| T1 | -.15 | .07 | .19 | .17 | .17 | **-.42** | -.17 | .15 | -.09 | **.25** | -.14 |
| T2 | -.05 | **.23** | .05 | .20 | -.05 | **-.31** | .00 | .08 | -.08 | **.22** | -.04 |
| T3 | .12 | .18 | .09 | **.34** | .11 | **-.48** | .11 | .11 | -.18 | **.31** | -.19 |
| Valuing diversity |  |  |  |  |  |  |  |  |  |  |  |
| T0 | -.01 | .01 | .14 | **.33** | .11 | **-.39** | .08 | .08 | .011 | -.05 | -.04 |
| T1 | .01 | .12 | .07 | **.23** | -.00 | **-.40** | -.10 | .05 | **.27** | -.15 | **.25** |
| T2 | .09 | **.23** | .19 | .22 | -.15 | **-.41** | .07 | .07 | .15 | -.07 | .08 |
| T3 | .05 | **.36** | .17 | **.31** | -.29 | **-.43** | .06 | .21 | .06 | .02 | .03 |
| Promoting employee voice |  |  |  |  |  |  |  |  |  |  |  |
| T0 | .15 | -.09 | .10 | **.30** | .24 | -.21 | -.21 | -.09 | .15 | -.11 | -.05 |
| T1 | .04 | .10 | **.23** | -.01 | .10 | **-.41** | -.6 | -.16 | .16 | .09 | .17 |
| T2 | .16 | **.25** | **.31** | .17 | .00 | **-.46** | -.15 | -.02 | .11 | -.03 | .05 |
| T3 | .19 | .16 | **.29** | **.34** | -.01 | **-.61** | .07 | .07 | .07 | -.04 | .10 |
| Creating commitment |  |  |  |  |  |  |  |  |  |  |  |
| T0 | -.02 | **.27** | .11 | **.45** | .15 | **-.26** | -.17 | -.11 | .02 | -.11 | -.21 |
| T1 | .03 | **.31** | .05 | .14 | .06 | **-.26** | -.14 | -.14 | .12 | .04 | .03 |
| T2 | **.23** | **.42** | **.26** | .17 | -.03 | **-.42** | -.16 | -.09 | .06 | **.24** | -.06 |
| T3 | -.02 | **.27** | **.27** | **.35** | -.10 | **-.42** | .06 | -.01 | -.01 | .07 | -.09 |
| Negotiating |  |  |  |  |  |  |  |  |  |  |  |
| T0 | -.02 | .21 | **.25** | **.34** | .13 | **-.40** | **-.24** | -.11 | .03 | .09 | -.07 |
| T1 | -.01 | .15 | **.46** | .02 | .05 | **-.26** | -.22 | -.22 | -.07 | -.09 | .07 |
| T2 | .10 | **.27** | .21 | .03 | -.04 | **-.33** | **-.25** | -.16 | -.08 | .05 | .03 |
| T3 | -.04 | **.27** | **.25** | **.28** | .04 | **-.72** | -.01 | .02 | .10 | .20 | .14 |
| Managing stress |  |  |  |  |  |  |  |  |  |  |  |
| T0 | -.03 | .13 | **.25** | **.30** | .01 | **-.58** | -.06 | -.06 | .19 | -02 | .20 |
| T1 | -.04 | **.28** | **.22** | .04 | -.08 | **-.35** | -.14 | -.14 | .14 | .00 | .19 |
| T2 | **-.23** | **.28** | **.34** | .02 | -.02 | **-.54** | -.11 | .04 | .12 | -.04 | .16 |
| T3 | -.20 | .18 | **.32** | .11 | -.02 | **-.62** | .18 | **.33** | .04 | -.07 | -.01 |
| Articulating complex ideas |  |  |  |  |  |  |  |  |  |  |  |
| T0 | .14 | -.01 | **-.24** | **.41** | .13 | **-.27** | .07 | -.08 | .07 | **.40** | -.08 |
| T1 | .03 | .15 | -.18 | .09 | .02 | -.15 | -.06 | -.13 | **.22** | **.25** | .01 |
| T2 | .08 | .12 | -.03 | .16 | -.08 | **-.27** | -.09 | -.09 | .12 | **.41** | .04 |
| T3 | -.08 | **.23** | -.05 | **.31** | -.05 | **-.53** | .15 | .15 | .08 | **.32** | -.03 |
| Adapting to change |  |  |  |  |  |  |  |  |  |  |  |
| T0 | -.02 | **.27** | **.30** | **.38** | .07 | **-.50** | .04 | -.19 | .04 | -.13 | -.06 |
| T1 | -.12 | .17 | **.26** | .20 | .06 | **-.38** | -.03 | -.23 | **.23** | -.15 | .06 |
| T2 | -.09 | **.28** | **.41** | .17 | .10 | **-.43** | -.15 | -.15 | .20 | -.07 | **-.26** |
| T3 | -.14 | **.43** | .20 | **.37** | .14 | **-.63** | .08 | -.01 | .08 | -.13 | -.10 |
| Promoted* | -.10 | .09 | .04 | .04 | .02 | .8 | -.03 | -.03 | **-.26** | -.04 | -.17 |
| Note: All coefficients in bold are significant at p<.05.  C - Coach | | | | | | | | | | | |

Supplementary Table 2: Means, standard deviations, and correlations of study variables

|  | LID0 | LID1 | LID2 | LID3 | SQ0 | SQ1 | SQ2 | SQ3 | VD0 | VD1 | VD2 | VD3 | PEV0 | PEV1 | PEV2 | PEV3 |
| --- | --- | --- | --- | --- | --- | --- | --- | --- | --- | --- | --- | --- | --- | --- | --- | --- |
| LID0 |  |  |  |  |  |  |  |  |  |  |  |  |  |  |  |  |
| LID1 | **.71** |  |  |  |  |  |  |  |  |  |  |  |  |  |  |  |
| LID2 | **.62** | **.65** |  |  |  |  |  |  |  |  |  |  |  |  |  |  |
| LID3 | **.58** | **.69** | **.74** |  |  |  |  |  |  |  |  |  |  |  |  |  |
| SQ0 | -.04 | **.24** | .10 | .06 |  |  |  |  |  |  |  |  |  |  |  |  |
| SQ1 | .05 | .22 | .21 | .15 | **.56** |  |  |  |  |  |  |  |  |  |  |  |
| SQ2 | .06 | .17 | **.27** | .12 | **.45** | **.53** |  |  |  |  |  |  |  |  |  |  |
| SQ3 | -.06 | .04 | .06 | .11 | **.50** | **.52** | **.61** |  |  |  |  |  |  |  |  |  |
| VD0 | .07 | .17 | .12 | .02 | **.47** | **.36** | **.45** | **.52** |  |  |  |  |  |  |  |  |
| VD1 | .17 | **.35** | **.35** | **.24** | .17 | **.39** | **.37** | **.36** | **.56** |  |  |  |  |  |  |  |
| VD2 | .05 | .11 | .24 | .12 | .20 | **.24** | **.44** | **.48** | **.55** | **.62** |  |  |  |  |  |  |
| VD3 | .06 | .11 | .12 | .07 | **.32** | **.26** | **.37** | **.49** | **.59** | **.53** | **.72** |  |  |  |  |  |
| PEV0 | -.00 | .15 | .06 | .09 | **.37** | **.33** | .08 | **.29** | **.41** | **.37** | **.28** | .14 |  |  |  |  |
| PEV1 | .02 | .16 | .24 | .10 | **.40** | **.54** | **.37** | **.33** | **.35** | **.50** | **.45** | **.24** | **.52** |  |  |  |
| PEV2 | .01 | .11 | .11 | .06 | **.26** | **.32** | **.44** | **.56** | **.33** | **.43** | **.61** | **.49** | **.41** | **.54** |  |  |
| PEV3 | .04 | .07 | -.03 | -.04 | **.30** | **.28** | **.29** | **.46** | **.42** | **.47** | **.58** | **.62** | **.32** | **.40** | .63 |  |
| CC0 | .14 | .19 | .12 | .23 | **.47** | **.45** | **.29** | **.48** | **.51** | **.34** | **.28** | **.38** | **.62** | **.27** | **.39** | **.31** |
| CC1 | .12 | .20 | **.25** | **.26** | **.28** | **.46** | **.46** | **.40** | **.28** | **.51** | **.38** | .22 | **.38** | **.63** | **.44** | **.32** |
| CC2 | .14 | **.26** | **.33** | .18 | **.34** | **.45** | **.43** | **.46** | **.30** | **.37** | **.50** | **.44** | **.33** | **.46** | **.66** | **.58** |
| CC3 | .16 | **.25** | .19 | .16 | **.38** | **.46** | **.60** | **.61** | **.44** | **.40** | **.50** | **.55** | **.33** | **.37** | **.59** | **.62** |
| N0 | .15 | .22 | **.37** | .16 | **.33** | **.33** | **.46** | **.45** | **.45** | **.40** | **.37** | **.34** | **.25** | **.37** | **.46** | **.34** |
| N1 | .08 | .17 | .20 | .16 | .13 | **.33** | **.34** | **.22** | **.39** | **.50** | **.43** | **.27** | **.26** | **.53** | **.49** | **.37** |
| N2 | .11 | .09 | .15 | .06 | .08 | **.29** | **.41** | **.41** | **.31** | **.44** | **.55** | **.36** | .15 | **.41** | **.58** | **.45** |
| N3 | .06 | .05 | .08 | .03 | **.26** | **.38** | **.41** | **.55** | **.42** | **.49** | **.57** | **.60** | .20 | **.38** | **.58** | **.65** |
| MS0 | .15 | **.35** | **.26** | **.27** | **.42** | **.41** | **.43** | **.47** | **.54** | **.61** | **.49** | **.49** | **.38** | **.45** | **.54** | **.57** |
| MS1 | .06 | .17 | .17 | .19 | .10 | **.35** | **.35** | **.31** | **.31** | **.48** | **.43** | **.32** | .20 | **.37** | **.52** | **.38** |
| MS2 | .11 | **.23** | .17 | .10 | **.32** | **.47** | **.47** | **.34** | **.35** | **.43** | **.39** | **.34** | **.24** | **.46** | **.57** | **.48** |
| MS3 | .07 | .18 | .15 | .05 | **.39** | **.29** | **.41** | **.46** | **.40** | **.30** | **.47** | **.47** | .09 | **.25** | **.46** | **.51** |
| ART0 | .05 | .05 | .12 | .03 | **.46** | **.40** | **.39** | **.42** | **.25** | **.22** | .18 | **.24** | **.35** | **.28** | **.29** | **.35** |
| ART1 | .13 | .14 | **.28** | .18 | .16 | **.40** | **.51** | **.25** | .06 | **.32** | .18 | .11 | .22 | **.40** | **.33** | **.23** |
| ART2 | .04 | .12 | .21 | .13 | **.36** | **.40** | **.42** | **.40** | .13 | **.23** | .18 | .09 | **.34** | **.37** | **.43** | **.24** |
| ART3 | .14 | .24 | .20 | .14 | **.41** | **.47** | **.64** | **.65** | **.32** | **.40** | **.45** | **.41** | **.23** | **.33** | **.51** | **.46** |
| AD0 | .15 | .21 | **.31** | .17 | **.42** | **.40** | **.42** | **.54** | **.57** | **.55** | **.56** | **.51** | **.38** | **.38** | **.48** | **.52** |
| AD1 | .22 | .19 | **.30** | .22 | .18 | **.44** | **.43** | **.22** | **.36** | **.57** | **.42** | .22 | **.43** | **.62** | **.40** | **.43** |
| AD2 | .15 | .21 | **.32** | **.28** | **.35** | **.54** | **.33** | **.34** | **.29** | **.46** | **.34** | **.34** | **.35** | **.50** | **.46** | **.48** |
| AD3 | .04 | .17 | .09 | .16 | **.47** | **.46** | **.44** | **.64** | **.51** | **.50** | **.58** | **.60** | **.35** | **.45** | **.57** | **.67** |
| Promoted* | -.03 | .03 | -.07 | -.01 | .06 | -.16 | .01 | .01 | -.11 | **-.31** | **-.35** | **-.28** | -.13 | **-.26** | -.20 | -.09 |
| Mean | 4.0 | 4.1 | 4.3 | 4.5 | 2.8 | 3.0 | 3.3 | 3.6 | 3.0 | 3.1 | 3.5 | 3.8 | 2.9 | 3.4 | 3.6 | 3.8 |
| SD | .56 | .61 | .57 | .49 | .81 | .74 | .75 | .80 | .81 | .78 | .75 | .78 | .96 | .89 | .87 | .72 |
| Note: All coefficients in bold are significant at p<.05. *Spearman’s (non-parametric) correlations coefficients in this row.  LID- leader identity, SQ - challenging the status quo, VD - valuing diversity, PEV - promoting employee voice, CC - creating commitment, N - negotiating, MS - managing stress, ART- articulating complex ideas, AD – adapting to change. | | | | | | | | | | | | | | | | |

|  | CC0 | CC1 | CC2 | CC3 | N0 | N1 | N2 | N3 | MS0 | MS1 | MS2 | MS3 | ART0 | ART1 | ART2 | ART3 | AD0 | AD1 | AD2 | AD3 | |
| --- | --- | --- | --- | --- | --- | --- | --- | --- | --- | --- | --- | --- | --- | --- | --- | --- | --- | --- | --- | --- | --- |
| CC0 |  |  |  |  |  |  |  |  |  |  |  |  |  |  |  |  |  |  |  |  | |
| CC1 | **.46** |  |  |  |  |  |  |  |  |  |  |  |  |  |  |  |  |  |  |  | |
| CC2 | **.41** | **.53** |  |  |  |  |  |  |  |  |  |  |  |  |  |  |  |  |  |  | |
| CC3 | **.43** | **.49** | **.73** |  |  |  |  |  |  |  |  |  |  |  |  |  |  |  |  |  | |
| N0 | **.40** | **.42** | **.49** | **.43** |  |  |  |  |  |  |  |  |  |  |  |  |  |  |  |  | |
| N1 | **.39** | **.56** | **.44** | **.39** | **.52** |  |  |  |  |  |  |  |  |  |  |  |  |  |  |  | |
| N2 | **.25** | **.34** | **.57** | **.47** | **.46** | **.63** |  |  |  |  |  |  |  |  |  |  |  |  |  |  | |
| N3 | **.39** | **.45** | **.56** | **.61** | **.52** | **.45** | **.52** |  |  |  |  |  |  |  |  |  |  |  |  |  | |
| MS0 | **.54** | **.49** | **.52** | **.54** | **.55** | **.52** | **.41** | **.59** |  |  |  |  |  |  |  |  |  |  |  |  | |
| MS1 | **.43** | **.45** | **.58** | **.51** | **.37** | **.55** | **.60** | **.48** | **.61** |  |  |  |  |  |  |  |  |  |  |  | |
| MS2 | **.37** | **.44** | **.56** | **.59** | **.48** | **.50** | **.51** | **.51** | **.62** | **.73** |  |  |  |  |  |  |  |  |  |  | |
| MS3 | .20 | **.23** | **.40** | **.51** | **.41** | **.25** | **.26** | **.52** | **.46** | **.40** | **.65** |  |  |  |  |  |  |  |  |  | |
| ART0 | **.44** | **.30** | **.32** | **.40** | **.26** | .10 | .00 | **.34** | **.41** | .20 | .20 | .12 |  |  |  |  |  |  |  |  | |
| ART1 | .18 | **.58** | **.47** | **.47** | .20 | **.25** | .20 | **.27** | **.36** | **.44** | **.46** | .13 | **.55** |  |  |  |  |  |  |  | |
| ART2 | **.24** | **.34** | **.55** | **.42** | **.25** | .15 | .19 | **.26** | **.32** | **.40** | **.50** | **.24** | **.56** | **.62** |  |  |  |  |  |  | |
| ART3 | **.32** | **.36** | **.53** | **.67** | **.36** | .12 | **.34** | **.49** | **.43** | **.41** | **.52** | **.57** | **.54** | **.51** | **.64** |  |  |  |  |  | |
| AD0 | **.52** | **.36** | **.52** | **.60** | **.62** | **.39** | **.50** | **.60** | **.54** | **.43** | **.39** | **.42** | **.36** | .09 | **.25** | **.46** |  |  |  |  | |
| AD1 | **.32** | **.61** | **.47** | **.49** | **.49** | **.56** | **.40** | **.41** | **.48** | **.49** | **.55** | **.33** | .21 | **.48** | **.36** | **.32** | **.57** |  |  |  | |
| AD2 | **.48** | **.50** | **.62** | **.63** | **.56** | **.53** | **.47** | **.49** | **.48** | **.53** | **.54** | **.41** | **.29** | **.38** | **.29** | **.46** | **.64** | **.65** |  |  | |
| AD3 | **.53** | **.43** | **.51** | **.66** | **.53** | **.44** | **.53** | **.72** | **.58** | **.53** | **.53** | **.54** | **.33** | .19 | .20 | **.61** | **.72** | **.50** | **.67** |  | |
| Promoted* | -.07 | -.17 | -.13 | -.07 | -.06 | -.12 | -.08 | **-.24** | **-.25** | -.11 | .01 | .05 | -.03 | -.05 | .08 | -.03 | -.13 | -.09 | -.09 | -.09 | |
| Mean | 2.9 | 3.1 | 3.3 | 3.5 | 2.9 | 3.3 | 3.2 | 3.7 | 3.2 | 3.5 | 3.6 | 3.9 | 2.8 | 3.0 | 3.3 | 3.5 | 2.9 | 3.1 | 3.4 | 3.8 | |
| SD | .96 | .88 | .82 | .86 | .86 | .79 | .67 | .71 | .94 | .83 | .74 | .81 | .76 | .82 | .82 | .75 | .79 | .88 | .70 | .69 | |
| Note: All coefficients in bold are significant at p<.05. *Spearman’s (non-parametric) correlations coefficients in this row.  LID- leader identity, SQ - challenging the status quo, VD - valuing diversity, PEV - promoting employee voice, CC - creating commitment, N - negotiating, MS - managing stress, ART- articulating complex ideas, AD – adapting to change. | | | | | | | | | | | | | | | | | | | | |  |
